# EDI OCT evaluation of choroidal thickness in Stargardt disease

**DOI:** 10.1371/journal.pone.0190780

**Published:** 2018-01-05

**Authors:** Andrea Sodi, Daniela Bacherini, Chiara Lenzetti, Orsola Caporossi, Vittoria Murro, Dario Pasquale Mucciolo, Francesca Cipollini, Ilaria Passerini, Gianni Virgili, Stanislao Rizzo

**Affiliations:** 1 Department of Surgery and Translational Medicine, Eye Clinic, Careggi Teaching Hospital, Florence, Italy; 2 Department of Genetic Diagnosis, Careggi Teaching Hospital, Florence, Italy; Massachusetts Eye & Ear Infirmary, Harvard Medical School, UNITED STATES

## Abstract

**Purpose:**

Choroidal thickness (CT) evaluation with EDI-OCT in Stargardt Disease (STGD), considering its possible association with some clinical features of the disease.

**Methods:**

CT was evaluated in 41 STGD patients and in 70 controls. Measurements were performed in the subfoveal position and at 1000 μm nasally and temporally. CT average values in STGD and in the control group were first compared by means of Student’s T test. Then, the possible association between CT and some clinical features was evaluated by means of linear regression analysis. Considered clinical parameters were: age, age on onset, duration of the disease, visual acuity, foveal thickness, Fishman clinical phenotype, visual field loss and ERG response.

**Results:**

Average CT was not significantly different between controls and STGD patients. In the STGD group the correlation between CT and age (r = 0.22, p = 0.033) and age of onset (r = 0.05, p = 0.424) was modest, while that of CT with disease duration (r = 0.30, p<0.001) was moderate. CT and foveal thickness were also significantly but modestly correlated (r = 0.15, p = 0.033).

**Conclusion:**

In our series average CT is not significantly changed in STGD in comparison with the controls. Nevertheless a choroidal thinning may be identified in the more advanced stages of the disease.

## Introduction

Stargardt disease (STGD) is the most common form of inherited macular dystrophy with an estimated prevalence of about 1:10000 [1]. It is characterized by the degeneration of photoreceptors and retinal pigment epithelium (RPE) in the macular area with a progressive loss of central visual function. Rarely the disease spreads to peripheral retina with a consequent impairment of side vision. The onset is usually in childhood or early adulthood but some late onset cases have also been reported [2, 3]. Fundus appearance usually presents with macular atrophic changes (sometimes with the typical “beaten bronze” aspect) often associated with the presence of flecks. Optical Coherence Tomography (OCT) scans usually shows a reduction of foveal thickness and a disruption of subfoveal outer retinal layers [4, 5] while autofluorescence imaging clearly outlines the flecks and the RPE atrophic changes at the posterior pole [6, 7, 8, 9]. The disease is usually transmitted with an autosomal recessive inheritance pattern and associated with pathogenic sequence variants of the ABCA4 gene [10, 11], coding for a visual cycle transport protein. Enhanced Depth Imaging (EDI) is an advanced OCT visualization which allows a more detailed examination of the choroid and the quantitative evaluation of some of its structures [12, 13, 14]. With EDI the quality of choroidal imaging is improved as the OCT imaging is placed closer to the eye so that the images acquisition focuses on the choroid instead on the retina. In the last years EDI-OCT approach has been used to investigate the choroid in several retinal disorders such as age-related macular de generation [15, 16], degenerative myopia [17] and central serous chorioretinopathy [18]. In the last years only a few studies reported on choroidal imaging in retinal dystrophies. Yeoh et al [19] investigated a few cases of different inherited retinal degenerations while other studies specifically evaluated choroidal abnormalities in larger series of retinitis pigmentosa reporting a decrease in choroidal thickness [20, 21, 22, 23]. Until today choroidal thickness in Stargardt disease have been rarely investigated and the few studies available in the literature report conflicting results. In a paper focusing on Indocyanine Green (ICG) Angiography in STGD [24] the Authors reported on SD-OCT evidence of intact choroid in STGD but did not provided quantitative data. More recently Nunes et al [25] found in STGD a small but significant increase in choroidal thickness in comparison with normal controls. On the contrary Adhi et al [26] and Vural et al [27] reported in STGD a reduction of mean subfoveal choroidal thickness. In the present paper we evaluated choroidal thickness with EDI-OCT in a significant series of STGD patients, considering the possible association of subfoveal choroidal thickness with some clinical features of the disease.

## Materials and methods

### Study population

Forty-one patients with a clinical diagnosis of STGD were included in the study. The patients were consecutively recruited through the Referring Center for Inherited Retinal Degeneration of the Eye Clinic, Careggi Teaching Hospital, Florence, Italy. Criteria for STGD phenotype included the following: appearance in the first or second decade of life; bilateral progressive central vision loss; macular atrophy/dystrophy; normal caliber of retinal vessels; absence of pigmented bone spicules. The presence of flecks was often detected at fundoscopy but it was not considered mandatory for the diagnosis. Fluorescein angiography was performed only in a small number of the patients because in most cases it was not clinically helpful for the diagnosis. Moreover all the STGD patients included in the study should have undergone molecular genetic testing showing at least two causative ABCA4 mutations. Molecular genetic analysis was always performed at the Genetic Diagnosis Laboratory of Careggi Universitary Hospital, Florence, Italy. The control group consisted of 70 healthy subjects. Only subjects with visual acuity of 20/20 (1.0) were included in the control group. They partly corresponded to patients resulted not affected by eye diseases that were referred to the Eye Clinic for routine examination. Exclusion criteria included relevant refractive errors (myopia higher than 5 dpts, hyperopia higher than 3 dpts and astigmatism higher than 3 dpts), significant cataract or other media opacities, ocular diseases other than STGD, age <15 or >40 years, and previous photorefractive or ocular surgery treatment. Moreover the patients did not show any significant systemic disease and none of them had a family history of other inherited retinal or systemic disorders. The study was approved by the local Institutional Review Boards (Careggi Teaching Hospital Ethics Committee) and adhered to the principles of the Helsinki declaration. Each patient included in the study signed a written informed consent to participate in this study, which was previously approved by the Ethics Committee.

### Ophthalmological examination

**Fig 1:**
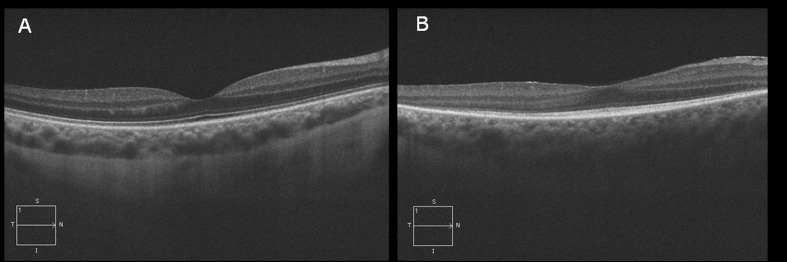
The pictures compare an EDI-OCT scan where choroidal borders could be reliably identified (A) with another scan with ill-defined choroidal borders (B).

A medical and ophthalmological history was first obtained from all the subjects included in the study. Then the patients underwent a comprehensive ophthalmological examination including visual acuity measurement with Snellen optotypes, evaluation of intraocular pressure with applanation tonometry, biomicroscopy of the anterior segment and fundoscopy after dilation with tropicamide 1% eye drops. In all the subjects SD-OCT scans were obtained with Cirrus Spectral Domain OCT (Carl Zeiss Meditec Inc., Dublin, CA, USA). The scan pattern used was the HRD Single Line Raster (providing the scan of a single high resolution line) with the EDI acquisition mode, allowing a detailed choroidal imaging. In the STGD group the acquisition protocol consisted also of a macular cube 512 x 128 scan pattern, to measure central retinal thickness. Images with a signal strength ≤9 were excluded. EDI-OCT black/white images were examined by manually measuring choroidal thickness using the specific cursor provided by the machine. Choroidal thickness (CT) was measured as the vertical distance from the hyperreflective line corresponding to the RPE and the hyperreflective line corresponding to the inner surface of the sclera. Measurements were performed in the subfoveal position and at 1000 nm nasally and temporally to the fovea, only in the cases where the border between choroid and sclera could be clearly identified (Fig 1A). The images where choroidal borders could not be clearly detected were excluded from analysis (Fig 1B). Because of the possible choroidal thickness variation at different times of the day [28, 29] all the subjects included in the study underwent EDI-OCT choroidal imaging during the morning clinic between 8.30 AM and 2 PM. Age of onset was considered corresponding to the first clinical diagnosis of the disease made by an ophthalmologist as in the first stages of the disease the mere subjective report of visual acuity reduction is difficultly distinguishable from an uncorrected refractive error. Duration of the disease was calculated from the difference between age and age of onset. Visual acuity was evaluated by means of Snellen charts and expressed in fraction, then converted in LogMAR for statistical analysis, while macular thickness was obtained by the OCT Macular Cube scan. Clinical phenotype was evaluated according to the Fishman classification distinguishing four stages of the disease [30]. The phenotype stage I was typically characterized by a localized atrophic appearing foveal lesion surrounded by perifoveal flecks. In stage II, the retinal flecks appear throughout the posterior pole, within the vascular arcades while in stage III there is a macular atrophy with an almost entire reabsorption of flecks. Finally in stage IV, there is an extensive atrophy of the RPE and choroid. Visual field severity grading was obtained classifying each eye in one of the following three levels of visual field alterations: central scotoma non exceeding the central 10° (stage 1), paracentral loss, extending beyond the central 10° (stage 2), diffuse perimetric abnormality including peripheral visual field loss (stage 3). According to Lois [31] ERG responses were classified as stage 1 if both standard scotopic and photopic tracings were normal, as stage 2 if only photopic response was impaired and as stage 3 if both scotopic and photopic alterations were detected. Clinical phenotype, OCT, visual field and ERG evaluation were always performed by three experienced operators (AS, OC, and DPM) who shared all the procedures; in case of disagreement the opinion of the senior observer (AS) was considered.

### Statistics

The average values of CT in the three considered locations (subfoveal, nasal, temporal) of STGD and control groups were first compared by means of Student’s T-test. Then the possible association between CT at the different locations and some clinical parameters was evaluated by means of mixed models accounting for within-subject correlation using a random effect. Clinical parameters considered in analyses were: age, age on onset, duration of the disease, visual acuity, foveal thickness, Fishman clinical phenotype, visual field loss and ERG response. Given the exploratory nature of the analyses, no adjustment for multiplicity of testing was performed. A statistical significance threshold of p< 0.05 was adopted. Pearson correlation was computed by means of Structural Equation Models (SEM) to account for between-subject correlation as a random effect at the individual level. We tested parameter group invariance among choroidal location (subfoveal, nasal, temporal) and we computed an average correlation coefficient if it was not shown to vary by location. All analyses were conducted using Stata 14.1 software (StataCorp, College Station, TX).

## Results

The study recruited 41 STGD patients (23 females and 18 males). All the patients carried at least two biallelic mutations of the ABCA4 gene. On average the age of the patients was 37.1 ± 12.2 yrs, the age of onset of the disease 22.1 ± 10.8 yrs and the disease duration 15.2 ± 11 yrs. Mean visual acuity was 0.96 ± 0.47 LogMAR (approximately Snellen value 20/200), while foveal thickness, automatically evaluated by the Cirrus OCT machine from the Macular Cube acquisition, was on average 82.7 ± 42.4 μm. In all the patients the clinical picture according to Fishman was the same in both eyes; specifically 42 eyes (21 patients) were classified as phenotype I, 18 eyes (9 patients) as phenotype II, 16 eyes (8 patients) as phenotype III, and 6 eyes (3 patients) as phenotype IV. Visual field was available in 35 patients (85%) and in all the patients the stage of visual field loss was the same in both eyes; fifty eyes (25 patients) could be classified as stage 1, 14 eyes (7 patients) as stage 2 and 6 eyes (3 patients) as stage 3. ERG was available in 24 patients (58%). Out of corresponding 48 eyes, 25 eyes could be identified as stage 1, 9 as stage 2 and 14 as stage 3. In the control group 70 healthy subjects (38 females and 32 males) were included in the study, with an average age of 40.1 ± 1.10 yrs. In this group average foveal thickness was 262.7 ± 18.7 μm.

In the STGD group, out of the 82 considered OCT scans we could reliably detect the choroidal borders in 71 eyes (87%) while the remaining 11 scans (14%) where choroidal limits were ill-defined were excluded from the study. The mean subfoveal CT was 286.7 ±84.4 μm while the mean CT at 1000 μm from the fovea was 260.2 ± 81.6 μm nasally and 275.2 ± 82.7 μm temporally. In the control group, choroidal borders could be reliably identified in 127 images (91%) which were considered for analysis, while the other 13 (9%) were not included in further evaluation processes. The mean subfoveal CT of the control group was 281.5 ± 67.0 μm, while the mean CT at 1000 μm from the fovea was 247.8 ± 65.8 μm nasally and 279.8 ± 65.6 μm temporally. Average CT was not significantly different between controls and STGD patients at all the considered positions (p>0.1). Averaging across positions, CT was non-significantly thicker in the STGD group in comparison with the controls by 9.4 μm (95%CI: -17.3 to 36.0 μm). Foveal retinal thickness was significantly reduced in the STGD group (82.7 μm ± 42.4 μm) compared to the control group (262.7 ± 18.7 μm, p <0.001).

### Association between CT and clinical and OCT variables in STGD

**Fig 2:**
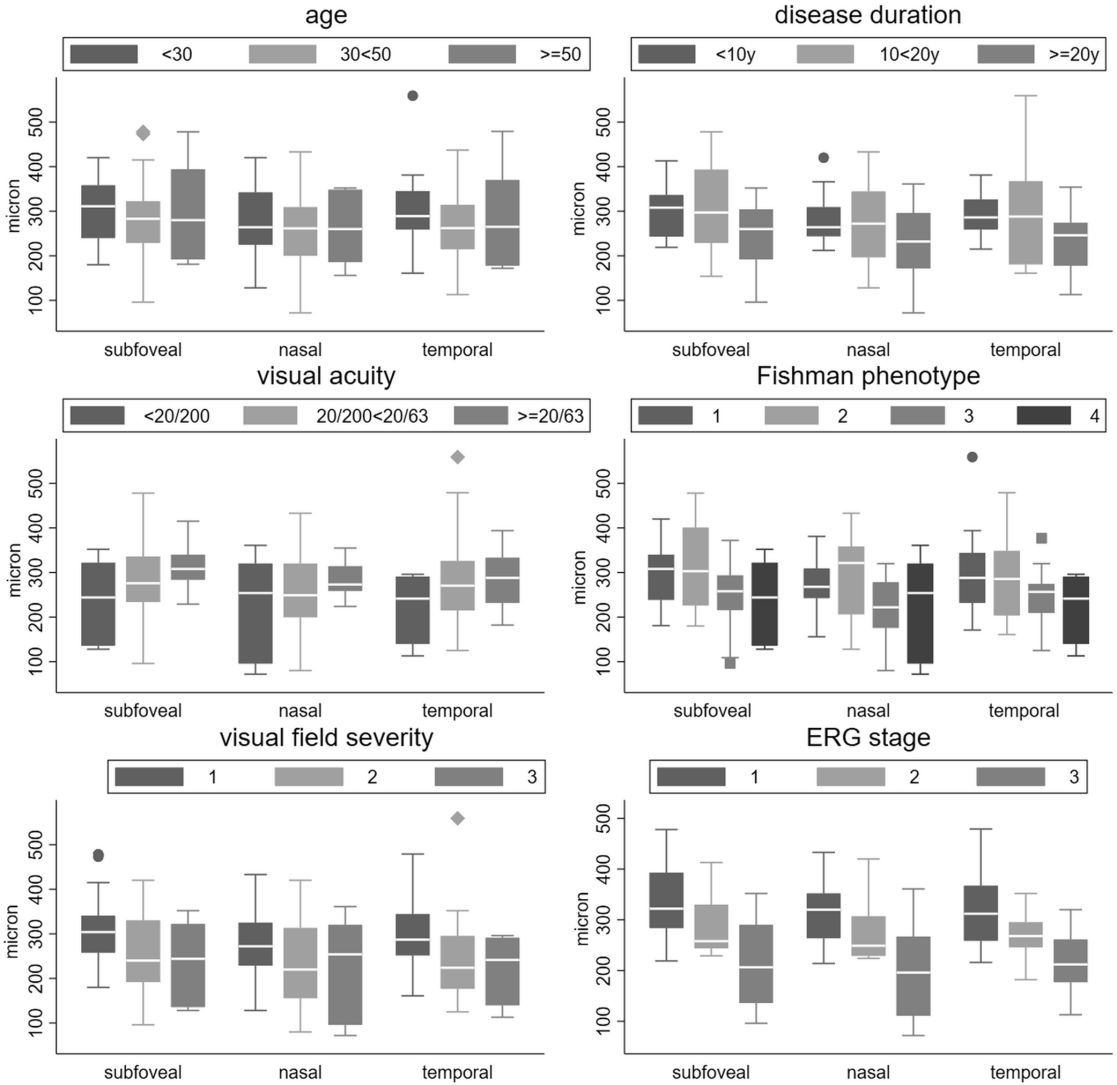
The graphs show the CT values across different subgroups of age (A), disease duration (B), visual acuity (C), as well as with Fishman phenotype (D), visual field severity stage (E) and ERG alteration grade (F).

In the STGD group the possible association of subfoveal, nasal and temporal CT with different clinical parameters was evaluated. Figs 2A–2F shows CT values across different subgroups of age (Fig 2A), disease duration (Fig 2B), visual acuity (Fig 2C), as well as with Fishman phenotype (Fig 2D), visual field severity stage (Fig 2E) and ERG alteration grade (Fig 2F). Because no evidence of differences of correlation coefficients among choroidal sites was found (p>0.1 for tests of group invariance in SEM) a single correlation coefficient was computed between choroidal thickness and each continuous variables, i.e. age, age of onset, disease duration and foveal thickness. The correlation between choroidal thickness and age (r = 0.22, p = 0.033) and age of onset (r = 0.05, p = 0.424) was modest, while that of CT with disease duration (r = 0.30, p<0.001) was moderate. Choroidal and foveal thickness were also significantly but modestly correlated (r = 0.15, p = 0.033). Similarly, we found no difference between choroidal sites and their association with categorical variables such as Fishman phenotype, visual field severity stage and ERG defect grade. Therefore, a single measure of association between CT and these variables was obtained as a test for linear trend of decreasing CT and severity stage of each categorical variable. CT decreased by -24 micrometer per each stage of Fishman phenotype (p = 0.049), -41 micron per stage of VF severity (p = 0.035) and -60 micron per stage of ERG alteration (p<0.001).

## Discussion

In the present research we reported on the evaluation of choroidal thickness (CT) by means of EDI-OCT in Stargardt disease (STGD). Forty-one STGD patients were recruited and their data were compared with those of an age- and sex-matched control group. A reliable identification of choroidal limits (especially the border between choroid and sclera) was possible in 87% of the STGD and in 91% of the control group. In all the patients included in the study choroidal thickness was measured in both eyes in the subfoveal position and 1000 μm nasally and temporally. For all the three considered measurement positions there was no statistically significant difference between STGD and controls even if in the nasal position CT was higher in the STGD group in comparison with the controls (260.2 μm vs 247.8 μm, respectively). So our research cannot support either the findings of a choroidal thickening in STGD reported by Nunes et al [25] or the results of Adhi et al [26] and Vural et al [27] who found in STGD a reduction of mean subfoveal choroidal thickness.

**Fig 3:**
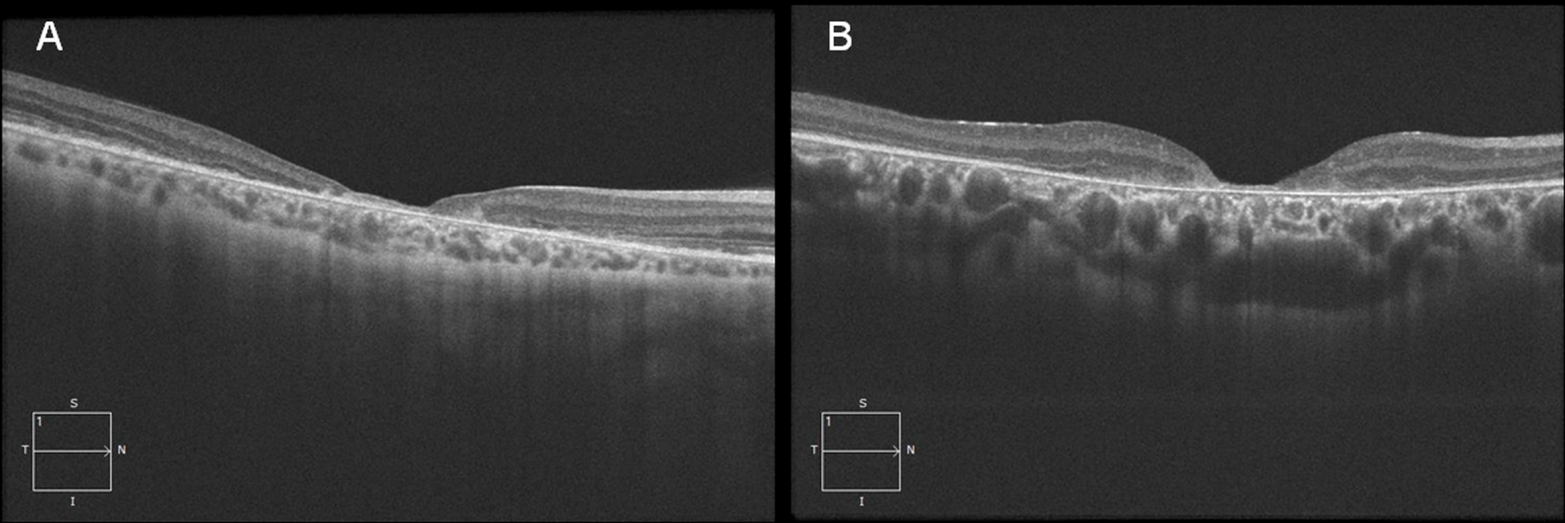
The pictures outline the variability of choroidal thickness in STGD. A) In this patient a thin choroid was associated with a Fishman phenotype 2, a relatively early onset (15 years) and a longer duration (11 years) of the disease. Visual acuity was 20/400, visual field loss consisted of paracentral scotomas and ERG photopic response was severely abnormal. B) In this patient a thick choroid was associated with a Fishman phenotype 1, a relatively late onset (25 years) and short duration (5 years) of the disease. Visual acuity was 20/200, visual field loss was limited to a central scotoma while ERG shows abnormal photopic response.

As our STGD patients may present a variable choroidal thickness (Fig 3A and 3B) we considered the possible association of CT with different clinical parameters of our population. Our data do not suggest an association of CT with age, and this may be due to the fact that we mostly recruited relatively young patients with an approximate mean age around 40 yrs. So the age variation is probably too limited to allow a significant influence of age on CT. On the contrary, in our STGD patients CT thinning is associated with a more severe clinical picture. In fact, even if the identified differences are not always statistically significant, CT is positively associated with a better visual acuity and a higher foveal thickness and is inversely related to duration of the disease, more severe fundus alterations (according to Fishman phenotypes), more advanced visual field loss and ERG abnormalities. These data are in agreement with those of Vural et al [27] reporting a statistically significant correlation between subfoveal CT and BCVA inner and outer retinal thickness and paracentral mf-ERG responses. They suggest that in STGD choroidal thickness is related to the progression of the disease and that the average results variability in the different studies may be due to the inclusion of STGD patients with more or less advanced stages of the disease. Moreover these results are in agreement with some preliminary investigations in a smaller series [19] reporting a variable choroidal involvement in STGD, ranging from no involvement to severe thinning. The Authors suggests that in STGD choroidal atrophy may occur at a later stage of the disease in association with a significant disruption and/or disappearance of RPE layer.

In STGD retinal pigment epithelium (RPE) is probably affected since the early stages of the disease and its alterations may interact with a progressive choroidal degeneration [32]. In fact RPE normal activity is crucial not only for choroid development but also for choroid proper function in adults as RPE destruction causes choriocapillaris atrophy [33]. Probably RPE performs its trophic function by secreting a variety of growth factors like transforming-growth factor β (TGF-β), Ciliary Neurotrophic Factor (CNTF), Pigment Epithelium derived Factor (PEDF) and mainly Vascular Endothelial Growth Factor (VEGF) [34, 35, 36]. Conversely choroid supplies nutrients and oxygen to RPE and outer retina and then choroidal changes may play a role in worsening the progression of RPE and photoreceptors degeneration in STGD. Similar hypothesis have been suggested for the role of the choroid in the pathogenesis of atrophic Age-related Macular Degeneration (AMD). In fact some Authors [37] found a thinner choroid in eyes with geographic atrophy and an histopathological study reports a linear relationship between RPE loss and choriocapillaris disappearance in atrophic AMD [38]. Nevertheless other studies did not confirm a variation of choroidal thickness in AMD [15, 16] or a correlation between choroidal thickness and AMD stages [39].

Giani et al [24] found with ICG-angiography that hypocyanescence from the areas of atrophy was more frequent in STGD compared with atrophic AMD. Moreover in their STGD series the choroid under the areas of atrophy seemed to be morphologically intact as showed by SD-OCT. The Authors interpreted these data as suggesting a possible selective damage of the choriocapillaris in STGD. In our study we did not directly compared choroidal thickness in STGD and AMD, nevertheless the two studies agree on the conclusion that in the overall STGD groups it was not possible to remark significant CT changes in comparison with the controls. Moreover, the two investigations are hardly comparable as the study of Giani et al [24] focused on ICG angiography did not provide a systematic quantitative evaluation of choroidal thickness and did not try to correlate their CT evaluation with clinical parameters related to the progression of the disease. Finally, due to the variability of STGD phenotype, the patients included in the two research groups may show a different spectrum of clinical pictures.

We are aware of some limitations of our study. First, the sample size was relatively small even if it was large enough to include a variety of patients with different clinical features and allow the detection of significant associations between CT and stage of the disease. Then CT measurement was performed manually by using the specific cursor provided by the OCT machine with an approach that remains operator-dependent. However the direct participation of trained operators may be useful to avoid possible mistakes determined by an automatic segmentation procedure. Finally, in our study CT was measured from SD-OCT obtained with EDI acquisition mode. It is possible that more sophisticated OCT technologies like the Swept Source OCT may allow a better visualization of choroidal borders and then a more accurate CT measurement.

In conclusion in our series STGD is not always associated with significant changes in choroidal thickness evaluated by means of EDI-OCT. Nevertheless a choroidal thinning may be identified in the more advanced stages of the disease probably in association with a severe RPE loss. EDI-OCT choroidal evaluation may be clinically useful for a prognostic evaluation of STGD and for a more refined selection of patients to be included in clinical trials.

## Supporting information

S1 Dataset Stargardt patients dataset. (XLSX)
